# Effects of Knotting on the Collapse of Active Ring Polymers

**DOI:** 10.1021/acs.macromol.5c02097

**Published:** 2025-09-30

**Authors:** Davide Breoni, Emanuele Locatelli, Luca Tubiana

**Affiliations:** † Department of Physics, Università di Trento, Via Sommarive 14, I-38123 Trento, Italy; ‡ INFN-TIFPA, Trento Institute for Fundamental Physics and Applications, I-38123 Trento, Italy; § Department of Physics and Astronomy, University of Padova, Via Marzolo 8, I-35131 Padova, Italy; ∥ INFN, Sezione di Padova, Via Marzolo 8, I-35131 Padova, Italy

## Abstract

We use numerical simulations to study tangentially active flexible ring polymers with different knot topologies. Simple, unknotted active rings display a transition from an extended phase to a collapsed one upon increasing the degree of polymerization. We find that topology has a significant effect on the polymer size at which the collapse takes place, with twist knots collapsing earlier than torus knots. Increasing knot complexity further accentuates this difference, as the collapse point of torus knots grows linearly with the minimum crossing number of the knot while that of twist knots shrinks, eventually canceling the actively stretched regime altogether. This behavior is a consequence of the ordered configuration of torus knots in their stretched active state, featuring an effective alignment for non-neighboring bonds which increases with the minimal crossing number. Twist knots do not feature ordered configurations or bond alignment, increasing the likelihood of collisions, leading to collapse. These results show that topology yields a degree of control on the properties of active ring polymers, and can be used to tune them. At the same time, they suggest that activity might introduce a bias for torus knots, as complex twist knots cannot be formed in extended active polymers.

## Graphical abstract

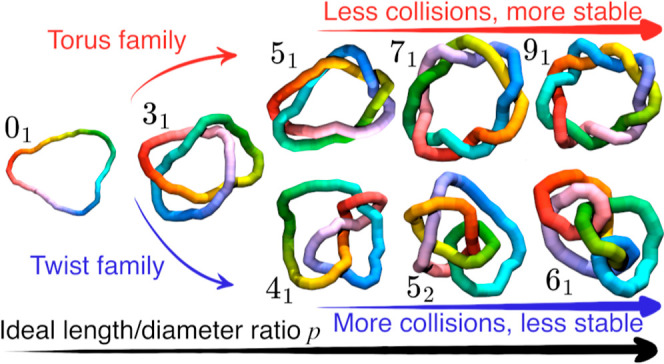

## Introduction

Polymers can attain a wide range of different topologies,^1^ from unknotted rings^2^ to knots and links,^3^ and from interconnected networks in melts^4,5^ to mechanically interlocked molecules.^6^ For example, actin has been tied into knots in vitro and knots have also been found in a small but important fraction of proteins.^7–9^ DNA can be organized in supramolecular structures that present various degrees of topological complexity, from chromatin loop networks during interphase^10^ to looped bottlebrush-like shapes during duplication,^11^ and from Olympic networks, typical of the Kinetoplast DNA (kDNA, the mitochondrial DNA of the parasites Trypanosomatids^12^) to knots.^13,14^ In fact, knots become extremely likely in long and confined polymers, including DNA,^15–17^ where they can impact its sequencing and behavior.^18–20^ More generally, knots have a large impact on polymers’ configurations,^21–24^ and show a complex dynamics^25–28^ inclusive of interknots interactions.^29,30^

A typical property of biological systems is activity, that is the ability of turning external energy into directed motion, a mechanism by which individual agents inject energy into the system, driving it out-of-equilibrium. It is responsible for a plethora of phenomena, such as self-organization,^31,32^ collective motion and flocking,^33,34^ spontaneous flow^35^ and clustering in absence of attractive forces (also known as motility-induced phase separation, or MIPS).^36,37^ Active polymers and filaments,^38^ the focus of much theoretical and experimental interest in the past few years, present an even richer phenomenology. Besides the aforementioned collective motion,^39–41^ self-organization^42–44^ and clustering,^45^ active filaments display pattern formation,^46^ enhanced long-time diffusion constant,^47,48^ anomalous rheological properties^49^ and giant melt elasticity^50^ that put them at the forefront of the research for dynamically tunable nanomaterials^51–55^ as well as for advanced macroscopic soft robots.^56^ These nanomaterials are, for the most part, based on biopolymers such as DNA,^52,53^ microtubules^55^ and actin,^51^ that are among the most relevant polymers in nature. Activity comes from molecular motors that, for actin and the other cytoskeletal filaments, allow to provide the cell with rigidity and motility, while, for DNA, play crucial roles during replication, transcription and protein production.^57^

The study of active polymers has recently revealed interesting, unexpected, interplay with topology. Dense suspensions of active diblock ring polymers, where activity is realized as an additional higher temperature, display glassy behavior,^58^ originating from a network of entanglements called deadlocks.^59^ In the case of tangential activity, where the self-propulsion of each monomer follows the local backbone conformation, it was shown that active polymers are much more efficient in forming knots than their passive counterpart^60,61^ and are consequently less prone to adsorption.^62^ Even in the case of simple, unknotted ring polymers, simulations showed a swelling-collapse transition at infinite dilution, driven by activity,^63^ while in the semidilute regime swollen rings self-organize in clusters.^42^

In this work we employ numerical simulations, performed over a wide range of polymerization degrees, to systematically characterize how knot topology affects the properties of active polymer rings, and in particular the swelling-collapse transition observed in ref 63. We focus on two knot families: double-helix torus knots and twist knots, of increasing complexity,^64,65^ up to 19 crossings, and compare their behavior with that of the unknot. We find that the knot family has a strong impact on the collapse, that becomes more evident with increasing knot complexity, to the point that sufficiently complex twist knots do not support an extended phase, while torus knots do. We rationalize this behavior by analyzing the properties of the extended conformations, quantifying the probability of collisions between beads that can lead to deadlocks, and by comparing the extended conformations to those of ideal knots.

The manuscript is organized as follows: in Sec. Methods we go over the details of the model and the simulations, in section Results we present our results regarding the phenomenology of collapse (section The Collapse Transition) and its causes (section Bead Collisions and Loop Formation) and finally in section Discussion we summarize and discuss our conclusions.

## Methods

We simulate ring polymers in bulk as closed Kremer–Grest chains of *N* beads^66^ in good solvent, whose topology (i.e., their knot) is set at the beginning of the run. We employ the simulation code LAMMPS,^67^ with a in-house modification for implementing the tangential activity. The beads have diameter σ, mass *m*, are in contact with a thermal bath with energy *k*_B_ *T* and follow Langevin dynamics with damping time τ_γ_ = 0.3 τ, where $\tau =\sigma \sqrt{m/k_{B}T}$ is the unit of time of the system. They interact with each other via a WCA potential

1 $$V_{WCA}\equiv \{\begin{array}{ll} 4\varepsilon \left[{\left(\sigma /r\right)}^{12}-{\left(\sigma /r\right)}^{6}\right]+\varepsilon & r\leq r_{c},\\ 0 & r>r_{c}, \end{array}$$

where *r* is the distance between monomers, $r_{c}=\sqrt[{6}]{2}\sigma$ and ε = 300*k*_*B*_ *T*. Within the polymers, consecutive beads attract each other with a FENE potential

2 $$V_{FENE}\equiv \{\begin{array}{ll} -\frac{1}{2}KR_{0}^{2}\;\ln\left[1-{\left(r/R_{0}\right)}^{2}\right] & r\leq R_{0},\\ +\infty & r>R_{0}, \end{array}$$

where *K* = 30ε/σ^2^ is the stiffness of the spring and *R*_0_ = 1.05σ is the maximum bond length. The large value of ε and small value of *R*_0_ were chosen to stiffen this FENE potential and hence prevent polymers from changing their topology.^63^ Each bead *i* is furthermore subject to a bending potential

3 $$V_{b,i}\equiv \kappa \left(1-t_{i-1}\cdot t_{i}\right)$$

where κ = *k*_B_ *T* is the bending energy and **t**_*i*_ ≡ (**r**_*i*+1_ – **r**_*i*_)/(|**r**_*i*+1_ – **r**_*i*_|) is the normalized tangent vector between neighboring monomers with position **r**_*i*_. Finally, activity is introduced in the system as a constant force pushing each bead *i* tangentially to the backbone of the polymer^68–70^

4 $$F_{a,i}\equiv F_{a}\left(t_{i-1}+t_{i}\right)$$

where *F*_a_ is the magnitude of the active force. We quantify the strength of activity in the system with the adimensional Péclet number, defined as $Pe\equiv F_{a}\frac{\sigma }{k_{B}T}$ : it was set to Pe = 0 for passive systems and Pe = 10 for active ones.

The simulations have a time-step of d*t* = 10^–3^ τ and run for an overall time of 10^6^ τ. As large active molecules tend to collapse and enter a glassy behavior, it becomes unpractical to improve the statistics by increasing the simulation times, as autocorrelation times diverge. To obviate this issue, we simulate instead multiple independent realizations of each molecule, 30 to be specific. Simulations of active polymers start from previously equilibrated passive configurations.

We consider polymers with 20 ≤ *N* ≤ 1024 and multiple different knot topologies: twist knots and torus knots. Twist knots can be made by repeatedly twisting an unknot around its center and finally clamping the two extremities together.^1^ Torus knots can be constructed by embedding a curve on a toroidal surface,^71^ and are completely defined by the number of windings *h* around the torus axis of rotational symmetry (the helices) and the number of windings *w* around the torus itself, leading to the naming convention $T_{h}^{w}$ . In this manuscript we focus in particular on double-helix^72^ torus knots, *h* = 2, reporting also the behavior of two *h* = 3 topologies for reference (see Supporting Information). The characterization of *T*_3_^w^ torus knots will however require a second, larger study.

Knot complexity is loosely captured by the minimal number of crossings, MCN, i.e. the minimum number of crossings with which a knot can be drawn on flat surface. For torus knots this follows the relation MCN = *w*(*h* – 1). While MCN provides an intuitive measure of knot complexity, and works intrafamily, the number of knots sharing the same value of MCN grows exponentially with it. For this reason in the following we also use the parameter $p=\frac{L}{d}$ , the ratio between the length and thickness of an ideal knot of a given topology.^64,65^ This parameter has the further advantage of connecting topological and geometrical properties, as it can be interpreted as the minimum length of rope needed to tie a specific knot.

We label all knots with MCN ≤ 10 using the common Alexander–Briggs (*A*–*B*) notation, where the main number indicates the MCN and the index differentiates between knots with the same MCN. For torus knots, this yields $T_{2}^{w}\equiv w_{1}$ (for example, $T_{2}^{9}\equiv 9_{1}$ ). High complexity torus knots with MCN > 10 are not present in the *A*–*B* frame, so we refer to them with the $T_{2}^{w}$ format. Although the trefoil knot $T_{2}^{3}\equiv 3_{1}$ is a member of both the torus family and the twist family, we decide to group it mainly with the former, as its active behavior has more in common with torus knots. For what regards chiral knots, we used both the left-handed and right-handed chiralities interchangeably, as we observed no relevant difference between the two versions.

## Results

### The Collapse Transition

We quantify the collapse of the polymers by measuring their gyration radius

$$R_{g}\equiv \sqrt{\left\langle \sum_{i=1}^{N}{\left(r_{i}-r_{cm}\right)}^{2}\right\rangle /N}$$

where **r**_cm_ is the position of the center of mass of the polymer and ⟨·⟩ represents the sample average over time and different molecules. We notice in Figure 1 that knotted polymers show the same qualitative behaviors as unknotted ones: in the passive case there is a single regime, *R*_g_ ∝ *N* ^ν^, with ν = 0.588 for our good solvent, while in the active one two different regimes emerge with increasing *N*. First, one finds a stretched regime *R*_g_ ∝ *N* ^*a*^, with *a* > ν, while a collapsed regime *R*_g_ ∝ *N* ^0.41^ appears at large enough values of *N*;^63^ the two regimes are separated by a collapse transition at *N*_C_. Looking at Figure 1b,c it becomes immediately apparent that this collapse transition happens at a value of *N*_C_ that is different for each knot, with torus knots (Figure 1b) being more resistant to collapse than twist knots (Figure 1c), and with this difference steadily increasing with complexity (i.e., as the MCN grows larger). At small enough values of *N*, the gyration radius of passive and active knots becomes comparable: this is due to the fact that, for each knot, there exists a minimum number of beads with which it can be tied, irrespectively of activity. This number is model-dependent but can be approximated by the smallest integer larger than the length/diameter ratio *p* of ideal knots.^65,72,73^ It was found by Grosberg et al.^64^ that this ratio has a determining effect on the gyration radius of passive polymers. In fact, in the scaling law *R*_g_(*N*, *p*) ∝ *N* ^ν^ *p* ^–ν+1/3^ (see Supporting Information), topology only appears as *p*. A similarly straightforward treatment for active polymers is not possible, and an in-depth understanding of the configurations of active polymers of different families becomes necessary to address the behavior of *R*_g_ and active collapse.

**Figure 1:**
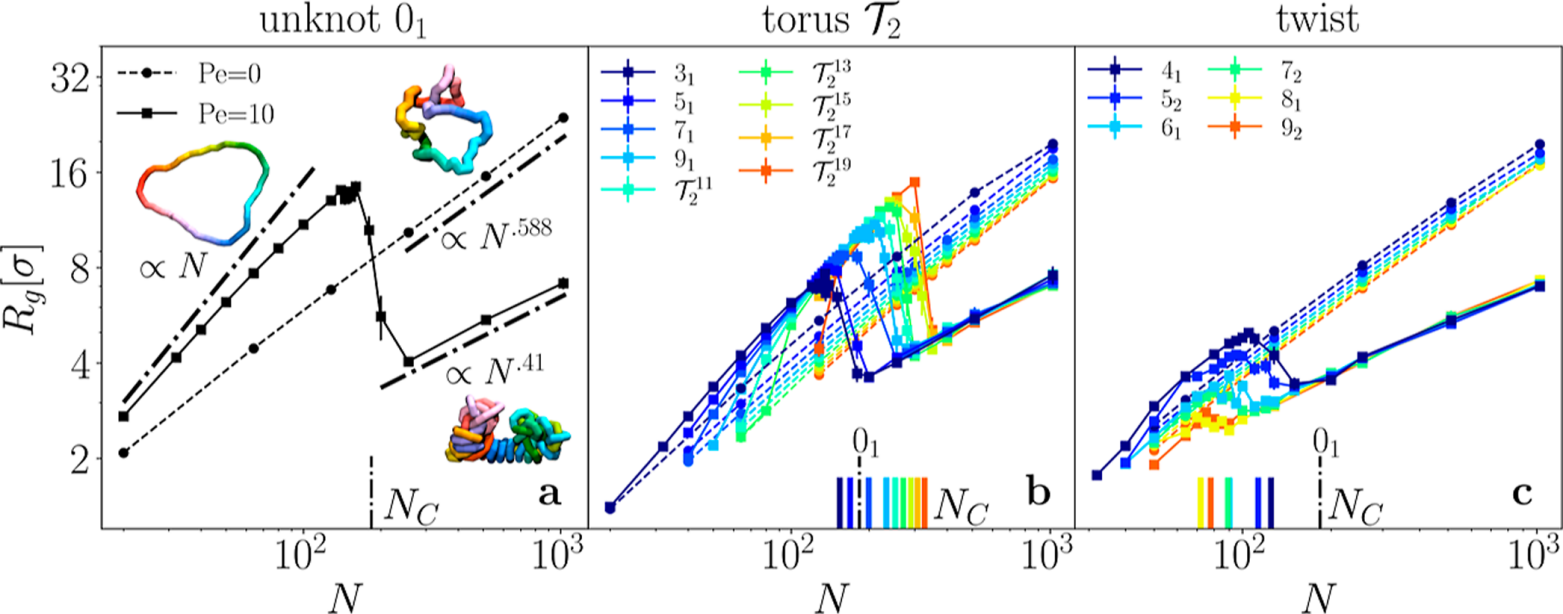
Gyration radius *R*_g_ of active (–) and passive (- -) ring polymers with various topologies: unknot 0_1_ (a) double-helix torus $T_{2}$ (b), and twist (c) knots as a function of polymerization *N*. The lines at the bottom represent the position of the collapse point *N*_C_, with the black longer dash-dot line representing *N*_C_ for unknots (estimation details in Supporting Information). The insets in (a) are simulation snapshots of unknots with different configurations: actively stretched (Pe = 10, *N* = 64, upper left corner), actively collapsed (Pe = 10, *N* = 256, lower right corner) and passive (Pe = 0, *N* = 64, upper right corner).

The effects of activity on polymer configuration, and especially their collapse, become apparent when studying the bond correlation function β­(δ) ≡⟨*∑*_(*i*,*j*)_ **t**_*i*_·**t**_*j*_/*N*⟩, where δ = |*i* – *j*| (see Figure 2). In the first regime, activity induces molecules to rotate at high speeds, as shown by a particularly large angular momentum density (see Supporting Information); this is accompanied by an effective increase in bending rigidity and long–distance correlation (Figure 2a,b,d, see Supporting Information for the passive case) which lead to stretched polymers and a larger *R*_g_. In the second regime instead, activity causes the total collapse of the molecules into twisted blobs whose bond orientation decorrelates after ≃ 5 beads (Figure 2c,e,f), reducing *R*_g_ significantly. The twisted conformations are particularly evident in the torsional parameter *U*_T_, defined as

5 $$U_{T}\equiv \left\langle \sum_{i}^{N}\frac{\left(t_{i-1}\times t_{i}\right)\cdot \left(t_{i}\times t_{i+1}\right)}{\left|t_{i-1}\times t_{i}∥t_{i}\times t_{i+1}\right|}\right\rangle$$

*U*_T_/*N* is large both when *N* is very small (close to *p*) and in the collapsed state, while polymers tend to minimize it in their actively stretched configurations (Figure 3). The collapse transition has furthermore a strong effect on the overall symmetry of the polymers, as stretched active polymers tend to be more oblate (disc-like) than collapsed polymers, with torus polymers being particularly asymmetric (see Supporting Information for a discussion on the asphericity and prolateness of the configurations). As already evident from Figure 1, topology becomes effectively irrelevant in the collapsed state, as *R*_g_ falls on the same master curve for all knots; crucially, we note that the collapse transition (delimited in Figure 2 by a red line) is strongly dependent on the specific topology of the polymer. In fact, all knots of the torus family (and the unknot) feature the transition at values greater than a certain threshold 135 ≲ *N*_*T*_ ≲ 145, while the twist knots collapse at lower values of *N*.

**Figure 2:**
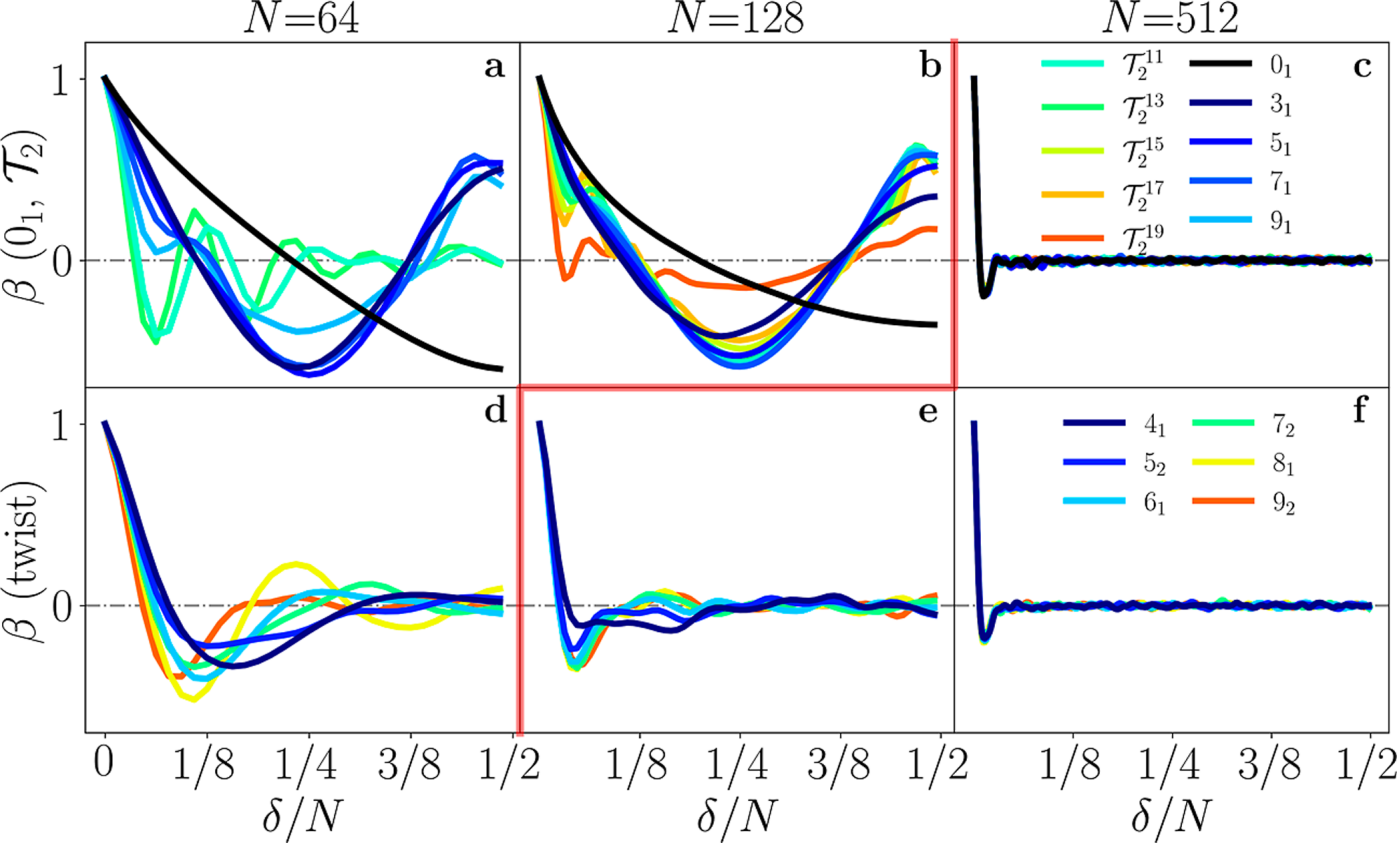
Bond correlation function β­(δ) of active ring polymers with various topologies: unknot 0_1_, torus $T_{2}$ (a,b,c) and twist (d,e,f) for different values of *N*. The red line remarks the collapse transition, which happens before 135 ≲ *N*_T_ ≲ 145 for all twist knots and after *N*_T_ for all $T_{2}$ ones. Within this phenomenology, trefoils and unknots behave as torus knots.

**Figure 3:**
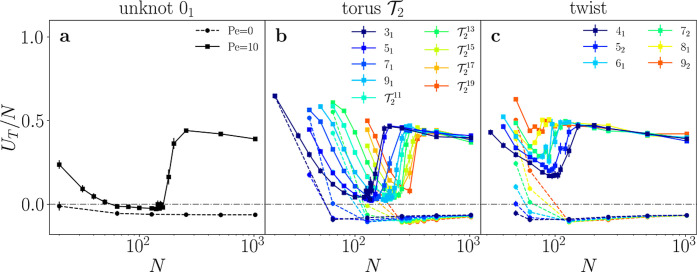
Torsional order parameter *U*_T_/*N* of active (—) and passive (- -) ring polymers with various topologies: unknot 0_1_ (a), double-helix torus $T_{2}$ (b), and twist (c) knots as a function of *N*.

Plotting the values of the collapse transition point *N*_C_ as a function of their corresponding ideal knot length/diameter ratio *p* (Figure 4a, see Supporting Information for details on the estimation of *N*_C_) highlights the qualitative difference between torus and twist knots, as *N*_C_ grows proportionally with *p* for torus knots, while it decreases for twist knots. This decrease is particularly significant, as it eventually leads to a complete disappearance of the actively stretched regime in twist knots. This phenomenon becomes more evident when one plots the excess length with respect to the length of the corresponding ideal knot, (*N*_C_ – *p*)­σ (Figure 4b). In doing so one can observe that the excess length decreases with *p* for twist knots, meaning that sufficiently complex twist knots can not maintain extended configurations, but directly collapse from their ideal configuration. This can be already seen for knots 8_1_ and 9_2_: their gyration radius growth in the stretched state appears as a small bump (Figure 1c), and we expect this regime to finally disappear for *p* ≳ 40, roughly corresponding to MCN ≳ 10. On the other hand, for torus knots *N*_C_ grows faster than *p* (Figure 4b), yielding a consistent stretched regime even at high complexity.

**Figure 4:**
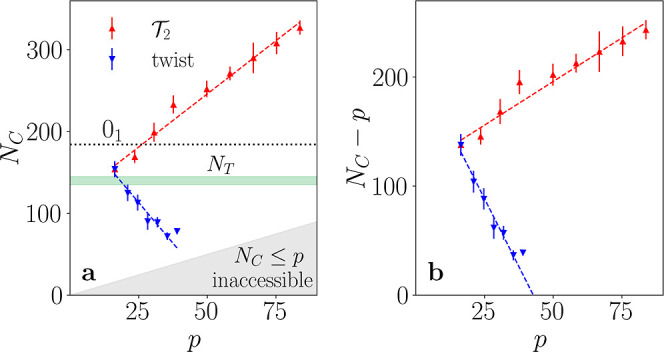
(a) Collapse point *N*_C_ for double-helix torus (red upper-facing triangles) and twist knots (blue downward-facing triangles) as a function of the ideal length/diameter ratio *p*. The trefoil knot is shown with both torus and twist symbols. The dotted black line represents the collapsing threshold for the unknot, while the dashed lines are meant to underline the behavior of *N*_C_ for the different families. The green area highlights that the collapse of all $T_{2}$ knots happens above *N*_*T*_, while that of all twist knots (except the trefoil) takes place below it. The gray area is inaccessible, as all knots must have a number of beads larger than *p*. See Supporting Information for the calculation of *N*_C_ and its uncertainty. (b) Difference between the collapse point *N*_C_ and *p* for torus and twist knots as a function of *p*. This difference determines the interval of polymer lengths at which the actively stretched state can exist: for torus knots this regime grows indefinitely, while for twist knots it shrinks with *p*, eventually becoming difficult to measure. The dashed lines are linear fits of the respective families, where the last twist knot (9_2_) was ignored because of the difficulty of measuring its *N*_C_.

### Bead Collisions and Loop Formation

To better understand the reason behind the difference in behavior between torus and twist knots, we measure how prone different systems are to bead–bead collisions. Collisions in colloidal active systems are known to trigger the formation of clusters at sufficiently high values of the Péclet number, in a process called motility-induced phase separation (MIPS).^36,37^ In melts of active rings, collisions of strands moving in different directions can also originate deadlocks,^59^ which prevent the molecules from relaxing and foster additional entanglements. Deadlocks in melt of rings are caused by one ring threading into another one, while in our case there is just a single strand passing inside a loop formed by another portion of the same ring. Still, a similar principle applies: due to activity this loop may get smaller and smaller, if the local conformation is strongly perturbed, thus stopping the motion of the strand, as it happens for unknotted rings.^63^ At the same time, the loop cannot be resolved, as it is topologically prevented by the strand passing through it. The stability of the resulting collapsed state could arise from the effective bending caused by activity, in a mechanism similar to that of passive tight knots in semiflexible polymers.^74–76^

We measure the probability of collisions by calculating the number density of close bonds (in 3D space) oppositely oriented ρ_3D_ ≡⟨(*#*{(*i*, *j*) ∈ *P*_*b*_: **t**_*i*_·**t**_*j*_ < 0})/*V*_*P*_⟩, where *V*_*P*_ = *N*4πσ^3^ is the volume of a tube of radius 2σ and length *N*σ. We only consider the set *P*_b_ of non-neighboring bonds (|*i* – *j*| > 3) within a distance in 3D space of 2σ from each other (|(**r**_*i*_ + **r**_*i*+1_)/2 – (**r**_*j*_ + **r**_*j*+1_)/2| < 2σ). This pair selection *P*_b_ is designed to measure the correlation of bonds **t**_*i*_·**t**_*j*_ in different sections of the polymer that come in contact with each other. In fact, as beads actively move in the direction of the local tangent to the polymer backbone, this orientational correlation contains information on intramolecular collisions, and more specifically tells us if two strands close to each other are likely to collide (null or negative correlation) or are moving in the same direction (positive correlation). Figure 5 shows how ρ_3D_ behaves as a function of *N*. In all cases ρ_3D_ initially decreases as the polymer extends further, reaches a minimum just before collapse and finally tends toward a value of around ρ_3*D*_ = 0.3σ^–3^. The value that this number density assumes before collapse, highlights the difference between families: torus and unknots reach smaller values than twist knots, and their strands have a consequently lower likelihood of colliding. Indeed, the minimum of ρ_3D_ is ≈0 for all torus knots considered, regardless of *p*. In contrast, in twist knots the likelihood of collisions increases with *p* (and therefore with MCN), as can be seen in Figure 5b.

**Figure 5:**
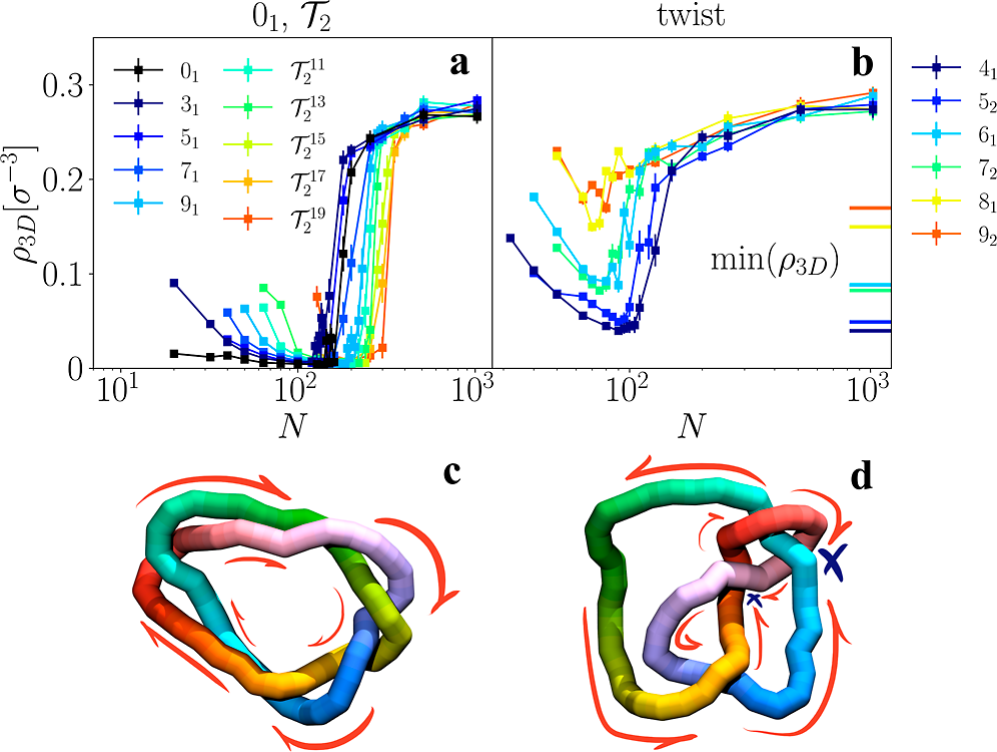
(a,b) Number density of close bonds oppositely oriented ρ_3D_ of active ring polymers with various topologies: unknot 0_1_, torus $T_{2}$ (a), and twist (b) knots as functions of *N*. All torus knots (and the unknot) present a vanishing probability of colliding strands in their stretched state, which is not the case for twist knots. The horizontal lines in (b) indicate the minimum value of ρ_3*D*_(*N*) for different twist topologies, showing that larger complexity twist knots have higher likelihood of colliding strands. (c,d) Snapshots of a 5_1_ torus knot (c) and a 4_1_ twist knot (d) in their actively stretched regime, with *N* = 64 and Pe = 10. The red lines sketch the direction of active motion, while the blue crosses represent points of collision. The regular conformation of the torus eases friction, while the twist knot presents multiple points of collisions, especially nearby its noose-like constraint.

While the behavior of ρ_3D_ sheds light on the mechanical reasons of the collapse, it still does not explain why torus knots are much less susceptible to collisions than twist knots, and also why the torus *N*_C_ grows linearly with *p*. The answer to these questions lies in the configuration of the stretched polymers, and especially their bond correlation β (see Figure 2). In particular, the point where the bond correlation β reaches its minimum, or arg min­(β), determines the typical loop size of the polymer *N*_*l*_ = 2⟨arg min­(β)⟩. We notice in Figure 2b that for the unknot β reaches a minimum around δ/*N* = 1/2, yielding a loop length *N*_*l*_ which almost coincides with *N*, as the full polymer turns into a loop (Figure 1a, top left). For $T_{2}$ knots the minimum is instead reached at δ/*N* = 1/4, compatible with a configuration resembling that of two loops intertwined in a double-helical shape (Figure 5c). This very regular shape, where strands moving actively in the same direction are intertwined, reduces ρ_3D_ to a minimum and makes the polymers less prone to collapsing.

When *N* grows, the pitch of the double helix grows as well, and with it the length of loops and the probability that some of them collide, collapsing the polymer. Increasing *p* lowers the helix’s pitch, and therefore the frequency of collisions. As a result, *N*_C_ increases on average by ≃ 20 beads with each pair of additional crossings. This length of 20 beads is particularly interesting, as it is the same one individuated by Locatelli et al. as the typical arg min­(β) at which active ring polymers starting in a passive state either extend further or collapse.^63^ Twist knots, on the other hand, show less regular configurations, and have no mechanism comparable to the one reducing ρ_3D_ in torus knots. On the contrary, they present noose-like constraints that constitute a systematical source for collisions and deadlocking (Figure 5d), with a consequently larger probability of collapse that further grows with the topological complexity, and hence *p*.

Finally, this picture allows to deepen our understanding of the collapse of the unknot. In their fully stretched state, unknots should presents few possibilities for collisions, as anticorrelated bonds are as far to each other as possible; yet, increasing *N*, the collapse transition is eventually observed. To clarify this, we looked into the typical conformations of active unknots just before their collapse transition; we noticed that they can assume a metastable folded configuration (Figure 6—top right, with an additional trajectory supplied in Supplemental video 1), which is very reminiscent of that of a stretched trefoil knot (Figure 6—bottom right). This comparison is further confirmed by the average crossing number, or ACN, defined as

6 $$ACN\left(C\right)=\frac{1}{4\pi }\int_{C}\int_{C}\left|\frac{r\left(s\right)-r\left(s^{*}\right)}{|r\left(s\right)-r\left(s^{*}\right){|}^{3}}\cdot \left[\frac{dr\left(s\right)}{ds}\times \frac{dr\left(s^{*}\right)}{ds^{*}}\right]\right|dsds^{*}$$

where *C* is a curve in 3D space. This quantity measures the average crossing number of a certain curve over all possible projections, and we can see in Figure 6 that the ACN for the unknot steadily grows with *N*, until reaching the value typical of an ideal trefoil curve around 140 < *N* < 160. This folded configuration increases the likelihood of collisions while remaining metastable, with respect to the extended state, as collisions between beads do not lead to full collapse. As *N* grows larger, more complex and less regular conformations will become available, leading to collapse.

**Figure 6:**
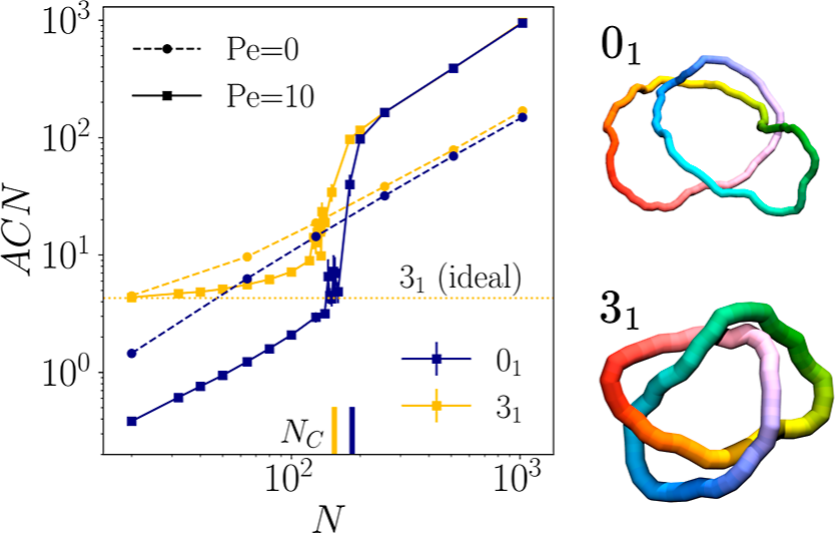
Average crossing number *ACN* for the unknot (blue) and the trefoil (yellow), both with activity (—) and without (- -); the dotted yellow line indicates the ACN for an ideal trefoil knot. To the top right of the image we show a simulation snapshot of an active unknot with *N* = 155, Pe = 10. This configuration is similar to that of a stretched trefoil, that can be seen in the bottom right (*N* = 64, Pe = 10).

## Discussion

In this manuscript we have studied how topology affects the collapsing behavior of tangentially active ring polymers. We found that torus $\left(T_{2}\right)$ and twist knots behave in opposing ways, as the collapse points *N*_C_ of the former family are all larger than those of the latter. Moreover, as the ideal knot length/diameter ratio *p* of the knots increases, the *N*_C_ of torus knots grows linearly, making them more resistant to collapse, while that of twist knots decreases, effectively erasing the stretched regime for *p* ≳ 40, or MCN ≳ 10. We identified the reason for this phenomenology in the configurations that different topologies take when actively stretched, and in the way these configurations foster or hinder bead collisions. In fact, we showed that torus knots tend to have regular double-helix configurations, with intertwined strands that move in the same direction, while twist knots present configurations where the presence of topological constraints, especially noose-like ones, acts as a catalyst for bead collisions, leading to MIPS-like clustering and deadlocking. Finally, the same arguments allows us to better understand the collapse transition in the unknot. Folded conformations appear in unknotted active rings with increasing *N*, close to *N*_C_: at first, these conformations maintain a regular structure and remain metastable with respect to the extended state. However, they introduce the possibility of collisions between strands: increasing *N* less regular arrangements become frequent, eventually leading to collapse.

In the future it will be important to test these phenomena in the presence of hydrodynamic interactions, as they have been shown to affect the configuration of ring polymers,^27,77^ although in the case of active polymers no qualitative difference has been observed yet.^78,79^ While this was currently unfeasible simply for the number of simulations required by a systematic characterization of torus and twist knots, we are confident that the qualitative message reported in this work will not change when hydrodynamics is explicitly considered, as the double-helix conformation of torus knots minimizes their energy and twist knots are topologically forced to have regions increasing the frequency of deadlock.

Our results widens the understanding of the interplay between activity and topology. First of all, they provide a way to control the extended regime of active polymers by tuning their topology.^80^ In fact, one could decide to produce polymers which collapse at arbitrarily large lengths by using torus topologies with enough crossings, or to completely suppress the stretched behavior by choosing instead a twist topology. The disappearance of extended twist knots also suggests that the knot spectrum could be used to detect the phase of randomly knotted active polymers, as complex twist knots would be exceedingly rare, or that activity could be used to select only torus knots.

Finally, this work could be extended to specific active materials where the activity and topology are both important. For example, it would be interesting to consider passive-active ring diblock polymers, testing the stability of active ring polymers as the percentage and distribution of active elements changes.^81^ This would also add on the picture given by the multiple studies on the formation of knots in linear diblock copolymers.^61,62^ Notably, molecular motors could effectively be modeled by tangential active beads moving along the backbone, pushing the polymer in the opposite direction. In such a case, knotted topologies would constitute an especially interesting playground, as molecular motors have been shown to also diffuse in 3D space,^82,83^ and the topological constraints would interplay and possibly facilitate such transport.

## Supplementary Material





## Notes

Calculation of the collapse point *N*_C_, additional properties of active knots, such as their angular momentum density *l*, asphericity *A* and prolateness *P*. Furthermore, gyration radius *R*_g_ and bond correlation function β for passive knots and behavior of the first two triple-helix $T_{3}$ knots (PDF)
Trajectory of an active unknot with *N* = 155, Pe = 10 in its metastable folded state (MOV)

## Notes

Preprint: A preprint of this manuscript is available on arXiv with the following DOI identifier: 10.48550/arXiv.2507.08391.
