# Supplementary material for: Effects of Knotting on the Collapse of Active Ring Polymers
Source: Macromolecules. 2025 Sep 30;58(19):10677–85. doi: 10.1021/acs.macromol.5c02097 (PMC12530043; doi:10.1021/acs.macromol.5c02097)
Supplement: Supplementary file 1 [file ma5c02097_si_001.pdf]

Effects of knotting on the collapse of active ring polymers – Supplemental Information

Davide Breoni,[*,†,‡] Emanuele Locatelli,[¶,§] and Luca Tubiana[†,‡]

†*Department of Physics , Università di Trento, Via Sommarive 14, I-38123 Trento, Italy*

‡*INFN-TIFPA, Trento Institute for Fundamental Physics and Applications, I-38123 Trento, Italy*

¶*Department of Physics and Astronomy, University of Padova, Via Marzolo 8, I-35131 Padova, Italy*

§*INFN, Sezione di Padova, Via Marzolo 8, I-35131 Padova, Italy*

E-mail: davide.breoni@unitn.it

Determination of the collapsing point N_C

To determine the collapse point N_C of an active polymer with a given knot, we use a special "cumulative" distribution of R_g, that is, the probability that the observed R_g is smaller than the average equilibrium value, $\mathcal{P}(N) \equiv P(R_g < 0.9\langle R_g^0(N)\rangle)$. We notice in fact that, before collapse, active knots generally feature $\langle R_g\rangle > \langle R_g^0\rangle$, while after the collapse the opposite holds. As such, the probability curve increases from 0 to 1 as N grows, allowing us to extract N_C by fitting this curve to a sigmoid function $\mathcal{S}(N) \equiv (1+\exp((N_C-N)/\eta))^{-1}$ (see Fig. S1). The sigmoid parameter η was used as uncertainty for the estimation of N_C. The 0.9 factor in the formula for $\mathcal{P}$ is necessary to keep $\mathcal{P}$ close to 0 for the small values of N at which R_g and R_g^0 tend to be very similar.

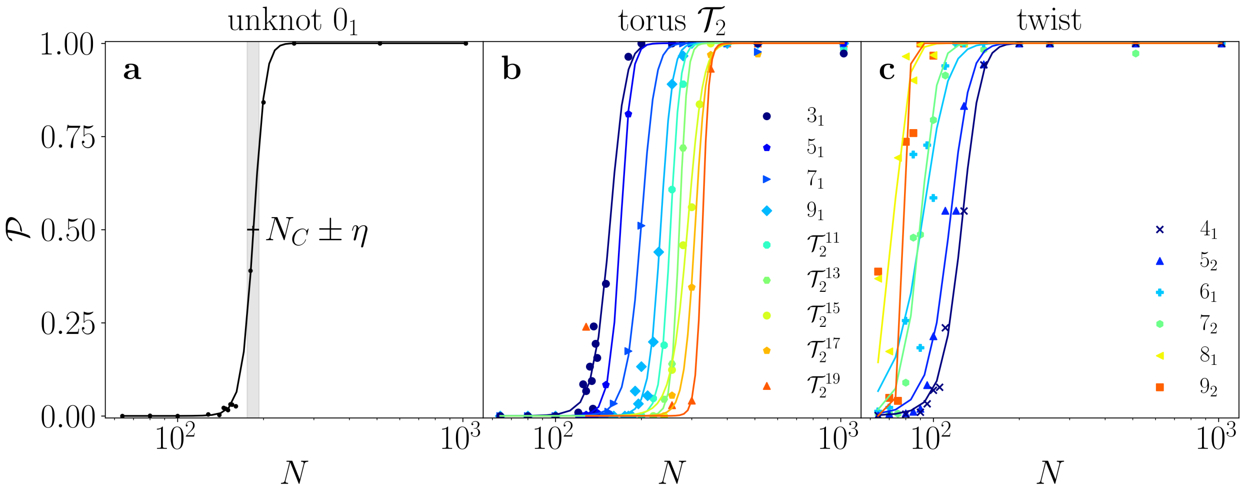

Figure S1: Probability $\mathcal{P} \equiv P(R_g < 0.9\langle R_g^0 \rangle)$ for the unknot (a), torus knots (b) and twist knots (c) as a function of N. The shaded area in (a) delimits the position and uncertainty of the collapsing point $N_C \pm \eta$.

Angular momentum density l

When in the stretched state, the configuration of the active polymers and the tangential forces allow them to rotate very fast around their normal axis. We measure this with the help of the angular momentum density l, defined as:

$$l \equiv \left\langle \frac{m}{N} \left| \sum_i^N (\mathbf{r}_i - \mathbf{r}_{cm}) \times (\mathbf{v}_i - \mathbf{v}_{cm}) \right| \right\rangle, \tag{1}$$

where m is the mass of the beads, $\mathbf{v}_i$ is the velocity of bead i, and $\mathbf{r}_{cm}$ and $\mathbf{v}_{cm}$ are respectively the position and velocity of the center of mass of the polymer. We notice that, for active polymers, l increases constantly until the collapse transition. Furthermore, unknots and $\mathcal{T}_2$ knots can reach significantly larger angular momenta than twist knots, as their monomers can rotate further away from the rotation axis (see Fig. S2).

Asphericity A and prolateness P

It was found by Rawdon et al.[1,2] that knot topology has an important effect on the conformation of passive knots, and more specifically the shape of their ellipsoid of inertia. Similarly,

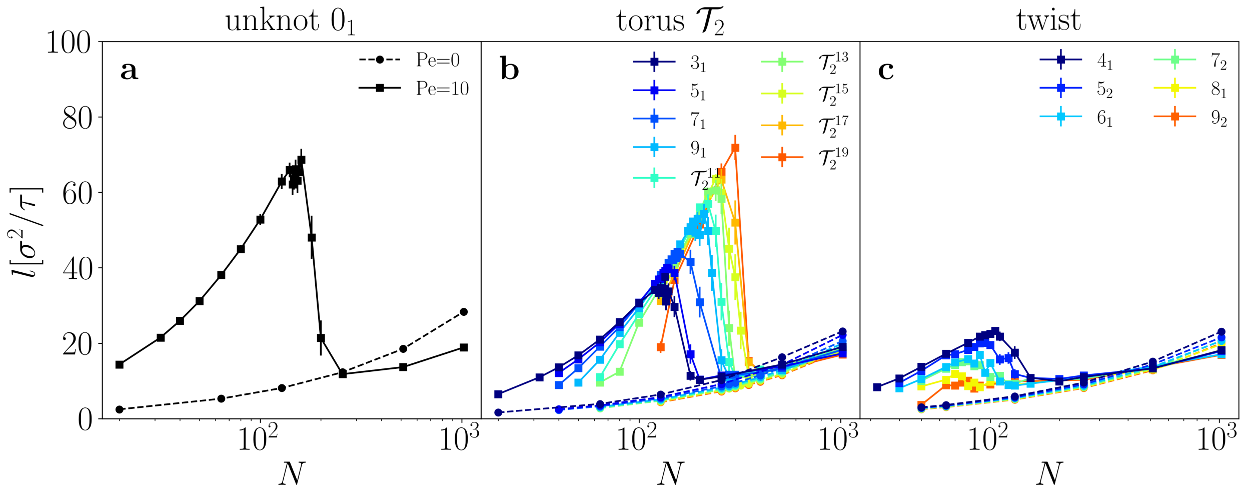

Figure S2: Angular momentum density l of active (—) and passive (- -) ring polymers with various topologies: unknot 0_1 (a), double-helix torus $\mathcal{T}_2$ (b), and twist (c) knot as a function of N.

we want to measure the effects of activity on the inertial ellipsoid of our polymers, and in order to do so, we calculate two quantities: the asphericity A, which gauges how spherically asymmetric the polymer is, and the prolateness P, which distinguishes between oblate objects (short and wide) and prolate objects (thin and long). Both quantities are computed starting from the moment of inertia tensor $T_{\alpha\beta}$, defined as

$$T_{\alpha\beta} \equiv \frac{1}{N}\left\langle \sum_{i=1}^{N}(r_i^\alpha - r_{cm}^\alpha)(r_i^\beta - r_{cm}^\beta)\right\rangle, \tag{2}$$

where $\mathbf{r}_{cm}$ is the center of mass of the polymer and α and β indicate the Cartesian axes. We then calculate the eigenvalues of $T_{\alpha\beta}$, whose square roots define the three semiaxes of the inertial ellipsoid: a, b and c. The asphericity A is then

$$A \equiv \frac{(a-b)^2 + (b-c)^2 + (c-a)^2}{2(a+b+c)^2}, \tag{3}$$

where $A = 0$ defines a spherically symmetric shape and $A = 1$ describes instead a rod-like shape. We observe that in their actively stretched state torus knots and unknots (Fig. S3c) are much more aspherical than twist knots (Fig. S3d) and their passive coun-

terparts (Fig. S3a,b). This is a result of the rather flat torus double-helix configurations.

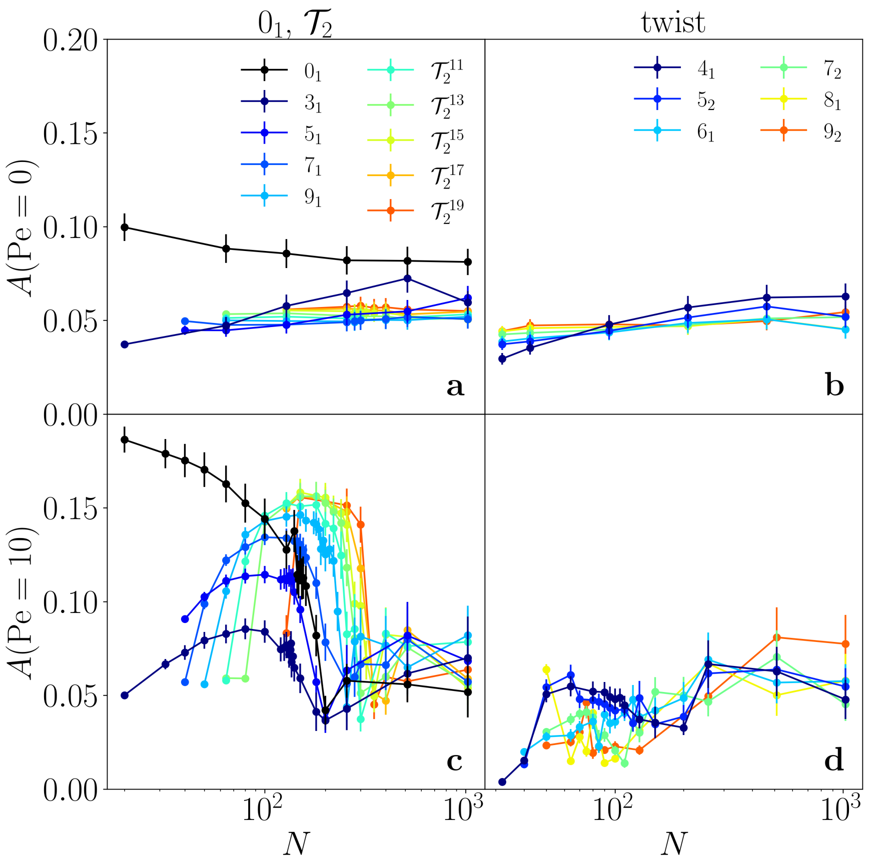

Figure S3: Asphericity A of passive (a,b) and active (c,d) ring polymers with various topologies: unknot 0_1, double-helix torus $\mathcal{T}_2$ (a,c), and twist (b,d) knots as a function of N.

The prolateness P is measured as

$$P \equiv \frac{(2a-b-c)(2b-a-c)(2c-a-b)}{2(a^2+b^2+c^2-ab-ac-bc)^{3/2}}, \tag{4}$$

going from $P=-1$ for an oblate disc-like object to $P=1$ for a prolate rod-like object. We notice that all active knots tend to go from an oblate state to a prolate one as they collapse (Fig. S4c,d), while the same is not always true for passive knots (Fig. S4a,b). Furthermore, active torus stretched configurations tend to be more disc-like than twist configurations.

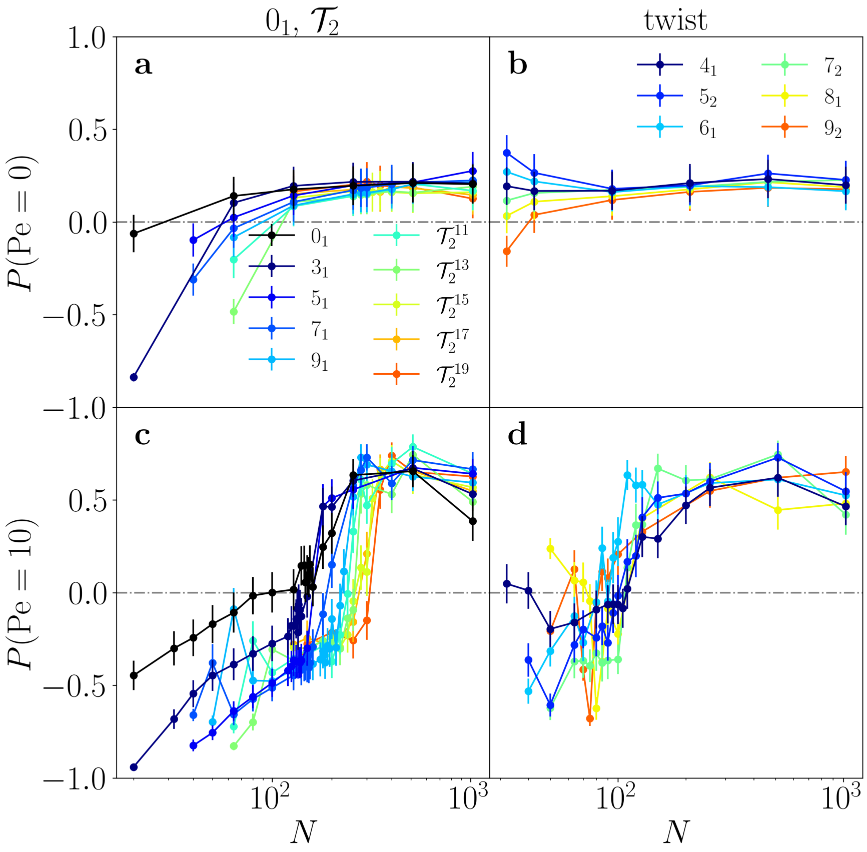

Figure S4: Prolateness P of passive (a,b) and active (c,d) ring polymers with various topologies: unknot 0_1, double-helix torus $\mathcal{T}_2$ (a,c), and twist (b,d) knots as a function of N.

Gyration radius and bond correlation function of passive polymers

In Fig. 1 of the main manuscript we see that the gyration radius R_g of passive ring polymers scales with N^ν, where $\nu = 0.588$, as typical of self-avoiding ring polymers in good solvent. Furthermore, we notice that R_g depends on the topology of the rings, decreasing as the complexity of the knot increases. This complexity can be quantified by taking the smallest length/diameter ratio p of the knot in its ideal configuration.[3–5] The dependence of R_g as a function of p was theoretically calculated by Grosberg et al. in Ref.,[6] yielding $R_g(N, p) \propto N^\nu p^{-\nu+1/3}$. We confirm this behavior in Fig. S5b, where R_g is rescaled accordingly, and as a result all knot topologies follow the same master curve. We exclude from this plot the unknot, for which p is not well defined.

To complete the information given by Fig. 2 of the main manuscript on the bond correlation

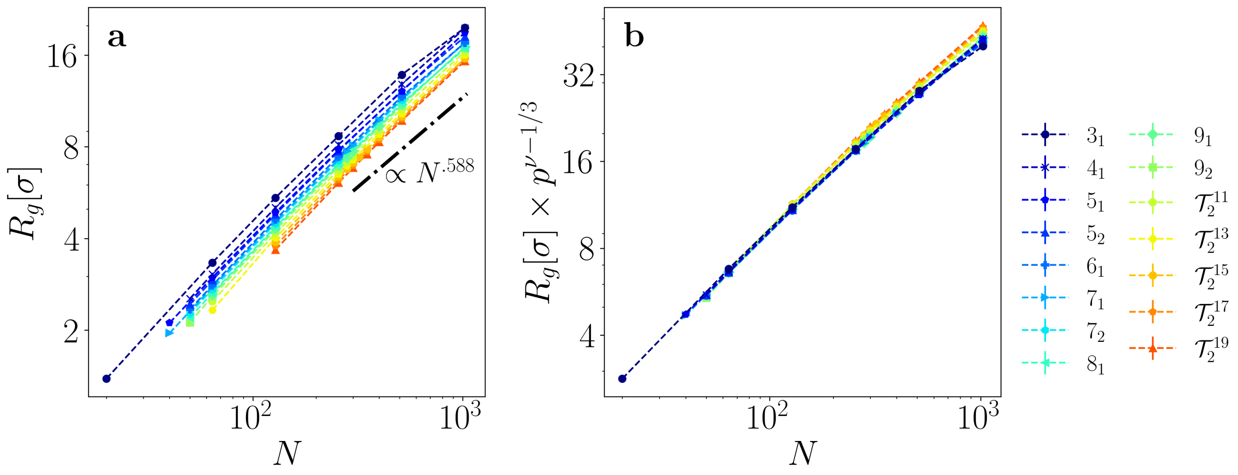

Figure S5: R_g of passive ring polymers with various topologies as a function of polymerization N (a) and rescaled to take into account knot complexity (b).

function, we show in Fig. S6 the results for passive systems with the same topologies and number of beads N. We notice that bending rigidity and anticorrelation are both weaker in the passive polymer case with respect to the actively stretched one.

Triple-helix torus knots $\mathcal{T}_3$

We studied the first two triple-helix torus knots $\mathcal{T}_3$ (8_{19} and 10_{124}), in order to assess whether they follow a behavior similar to that of $\mathcal{T}_2$ knots, and if so, to what extent. We see in Fig. S7 that both knots have a collapsing point close to that of the trefoil knot (Fig. S7a,b), with a slight increase in the N_C as p increases, which is more compatible with the behavior of $\mathcal{T}_2$ knots than with that of twist knots. This similarity is reinforced by the very regular shape of their bond correlation function (Fig. S8a), having for both knots a minimum at $N/6$, and by the rather small ρ_{3D} in the stretched state (Fig. S8b). As these preliminary results only consider the first two $\mathcal{T}_3$ knots though, no final conclusion should be made on their overall behavior.

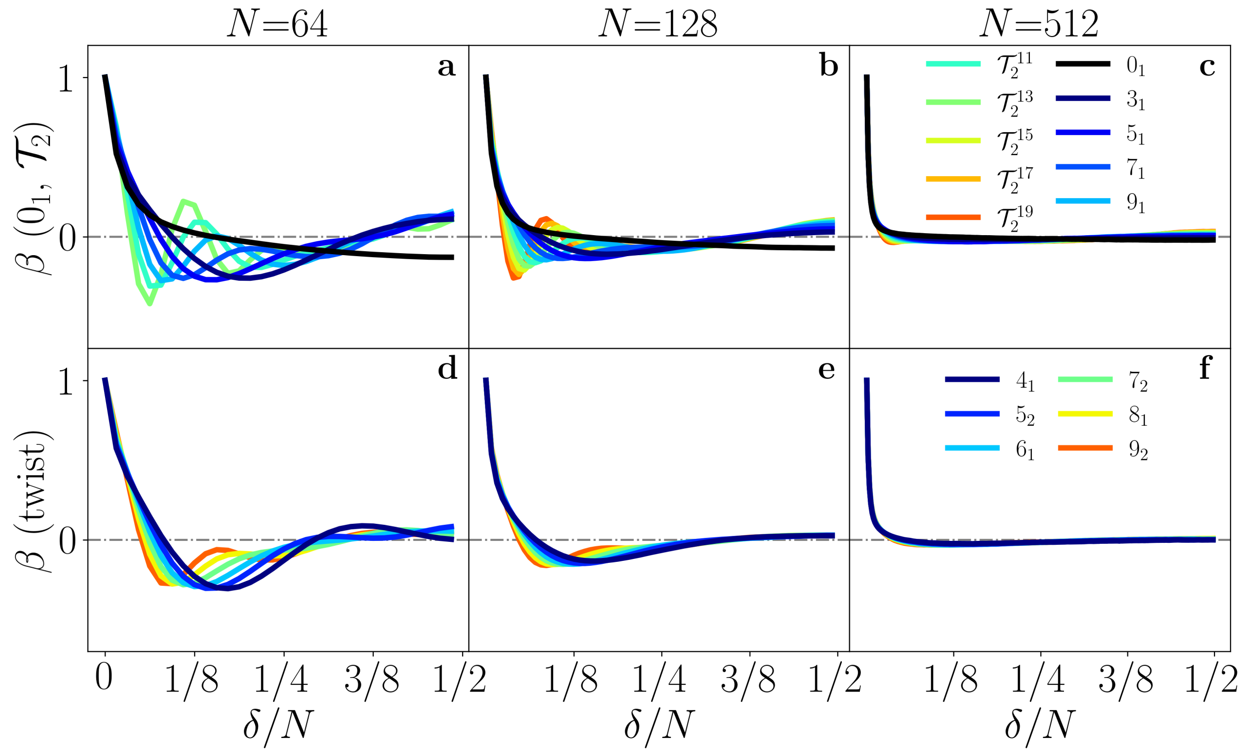

Figure S6: Bond correlation function $\beta(\delta)$ of passive ring polymers with various topologies: unknot 0_1, torus $\mathcal{T}_2$ (a,b,c) and twist (d,e,f) for different values of N.

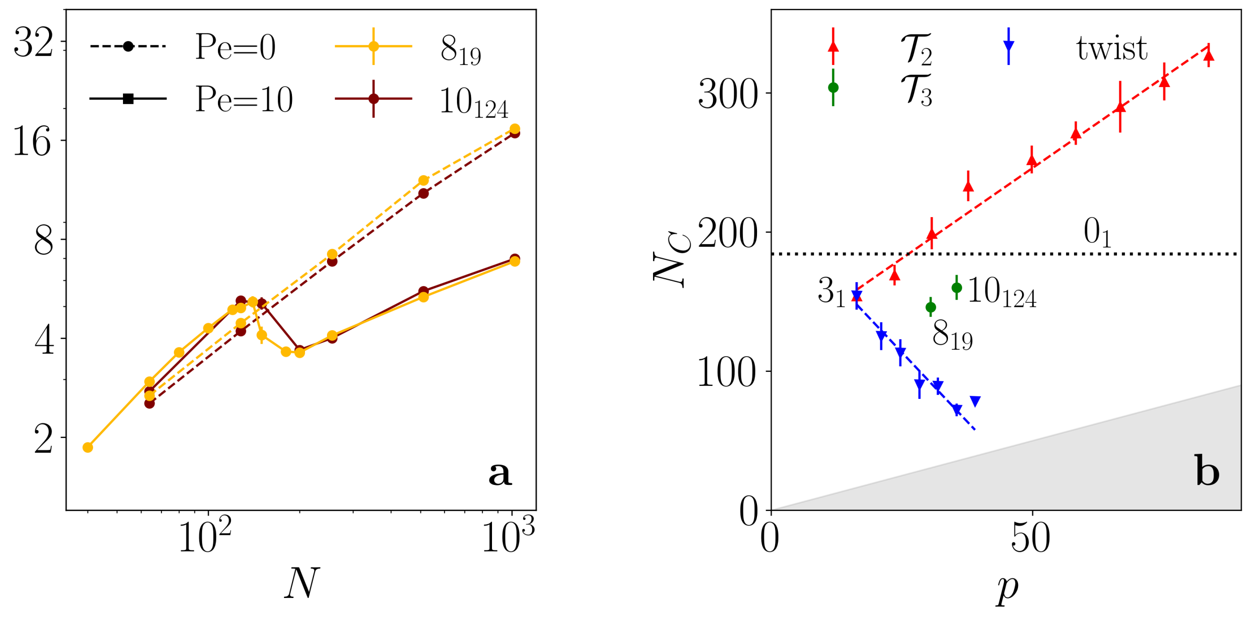

Figure S7: (a) Gyration radius R_g of passive (- -) and active(—) $\mathcal{T}_3$ knots, as a function of the polymer length N. (b) Collapse point N_C of $\mathcal{T}_2$ (red upward triangles), $\mathcal{T}_3$ (green circles) and twist knots (blue downward triangles) as a function of the ideal length/diameter ratio p

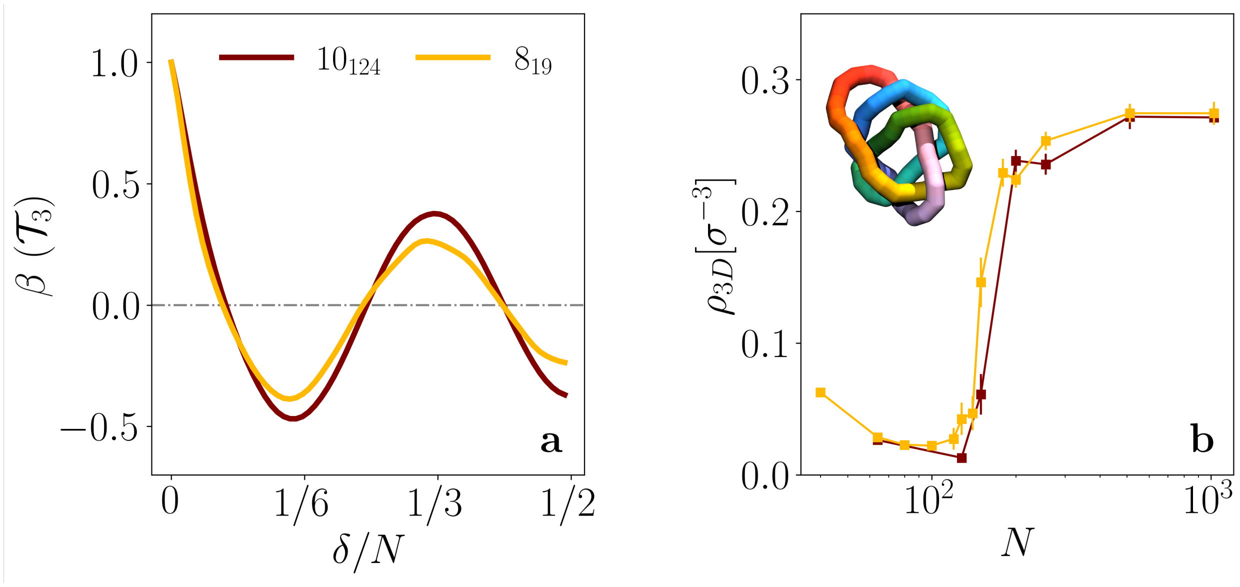

Figure S8: (a) Bond correlation function $\beta(\delta)$ of active ring $\mathcal{T}_3$ polymers for $N = 128$. (b) Number density of close bonds oppositely oriented $\rho_{3\mathrm{D}}$ of active ring $\mathcal{T}_3$ polymers as a function of the polymer length N. The inset is a simulation snapshot of an 8_{19} knot with $\mathrm{Pe} = 10$, $N = 64$.

References

(1) Rawdon, E. J.; Kern, J. C.; Piatek, M.; Plunkett, P.; Stasiak, A.; Millett, K. C. Effect of Knotting on the Shape of Polymers. *Macromolecules* **2008**, *41*, 8281–8287, DOI: 10.1021/ma801389c.

(2) Millett, K. C.; Plunkett, P.; Piatek, M.; Rawdon, E. J.; Stasiak, A. Effect of knotting on polymer shapes and their enveloping ellipsoids. *The Journal of Chemical Physics* **2009**, *130*, 165104, DOI: 10.1063/1.3117923.

(3) Katritch, V.; Bednar, J.; Michoud, D.; Scharein, R. G.; Dubochet, J.; Stasiak, A. Geometry and physics of knots. *Nature* **1996**, *384*, 142–145, DOI: 10.1038/384142a0.

(4) Stasiak, A.; Katritch, V. *Ideal Knots*; World Scientific, 1998.

(5) Olsen, K. W.; Bohr, J. A principle for ideal torus knots. *Europhysics Letters* **2013**, *103*, 30002, DOI: 10.1209/0295-5075/103/30002.

(6) Grosberg, A. Y.; Feigel, A.; Rabin, Y. Flory-type theory of a knotted ring polymer. *Physical Review E* **1996**, *54*, 6618–6622, DOI: 10.1103/PhysRevE.54.6618.
